# Pumpless extracorporeal lung assist in a pregnant woman with severe ARDS

**DOI:** 10.1186/cc10191

**Published:** 2011-06-22

**Authors:** HFRD Cunha, AP Coscia, A Longo, L Campioni, EG Martins, ED Meis, F Saddy, R Costa

**Affiliations:** 1 Hospital Quinta D'Or, Rio de Janeiro - RJ, Brazil; 2 Instituto Nacional do Cancer - INCA, Rio de Janeiro - RJ, Brazil; 3 Hospital Copa D'Or, Rio de Janeiro - RJ, Brazil

## Introduction

Acute respiratory distress syndrome is characterized by acute-onset, refractory hypoxemia, bilateral infiltrates on chest radiographs and PAOP <18 mmHg or absence of clinical signs of left atrial hypertension. The protective ventilatory strategy limiting plateau pressure to lower than 28 cmH_2_O, driving pressure below 15 cmH_2_O and tidal volume between 4 and 6 ml/kg using a PEEP level to sustain the open lung approach usually results in hypercapnia. However, it is the mainstream supportive therapy that can modulate survival in this syndrome.

## Methods

We describe a case report where a 31-year-old woman who was admitted to the intensive care unit with fatigue, shortness of breath and hypoxemia. She was 24 weeks pregnant and acute myeloid leukemia, subtype M3 was diagnosed 5 days before admission. Non-invasive ventilatory support, chemotherapy (doxirubicin and all-trans retinoic acid) and blood components (red blood cells, fresh frozen plasma, cryoprecipitate and platelets) were implemented. After 4 days the clinical scenario was out of control and she was intubated. Renal function deteriorated and hemodialysis was required.

## Results

Controlled mechanical ventilation using neuromuscular blocking (NMB) agents was set to limit plateau pressure, driving pressure, tidal volume and high level of PEEP (15 cmH_2_O). However, oxygenation progressively deteriorated despite the instituted therapy and on the eighth day on mechanical ventilation the intraabdominal pressure (IAP) was 20 mmHg, the driving pressure was 20 cmH_2_O and Vt was 5 ml/kg, which resulted in PaO_2_/FiO_2 _of 90, pH 7.15, PaCO_2 _of 115 mmHg. Interventional lung assist (iLA; Novalung, GmbH, Talheim, Germany), a pumpless arterio-venous extracorporeal membrane for CO_2 _removal, was connected without systemic anticoagulation. After 20 minutes using iLA with 9 l/minute O_2_, a PEEP level of 20 cmH_2_O, Vt of 4 ml/kg, driving pressure of 20 cmH_2_O, I:E of 1:1 resulted in a PaO_2_/FiO_2 _of 175, PaCO_2 _of 57 mmHg and pH 7.35. Hemodynamics were stable and vasopressor agents were not needed. The blood flow in the circuit was 1.4 l/minute. After 14 hours on iLA the NMB agent was interrupted and assisted ventilatory support with Bivent + PSV (Servo i Maquet, Solna, Sweden) was started, sustaining a driving pressure of 15 cmH_2_O. After 48 hours on iLA the baby was born naturally and the IAP decreased to 7 mmHg. Respiratory system mechanics and the PaO_2_/FiO_2 _ratio improved: 56% and 64%, respectively. CPAP + PSV was started on day 8 after iLA implementation and it was surgically removed on the day after when the PaCO_2 _was sustained below 40 mmHg.

## Conclusion

We present the first case so far where iLA was safely used during 9 days in a pregnant woman with severe ARDS and multiple organ dysfunction syndrome under continuous hemodialytic support that allowed us to set a protective ventilatory strategy using an assisted ventilation mode.

